# Acute Stress Exposure Alters Food-Related Brain Monoaminergic Profiles in a Rat Model of Anorexia

**DOI:** 10.1093/jn/nxab298

**Published:** 2021-09-14

**Authors:** Carter H Reed, Ella E Bauer, Allyse Shoeman, Trevor J Buhr, Peter J Clark

**Affiliations:** Interdepartmental Graduate Program in Nutritional Sciences, Iowa State University, Ames, IA, USA; Department of Kinesiology, Iowa State University, Ames, IA, USA; Interdepartmental Graduate Program in Nutritional Sciences, Iowa State University, Ames, IA, USA; Department of Food Science and Human Nutrition, Iowa State University, Ames, IA, USA; Neuroscience Program, Iowa State University, Ames, IA, USA; Department of Food Science and Human Nutrition, Iowa State University, Ames, IA, USA; Department of Food Science and Human Nutrition, Iowa State University, Ames, IA, USA; Neuroscience Program, Iowa State University, Ames, IA, USA; Interdepartmental Graduate Program in Nutritional Sciences, Iowa State University, Ames, IA, USA; Department of Food Science and Human Nutrition, Iowa State University, Ames, IA, USA; Neuroscience Program, Iowa State University, Ames, IA, USA

**Keywords:** stress, hypophagia, anorexia, eating behavior, monoamines

## Abstract

**Background:**

Adverse life experiences are a major risk factor for anorexia nervosa (AN). Eating-provoked anxiousness associated with AN is postulated to be due to food-related exaggerated serotonin activity in the brain and imbalances of monoamine neurotransmitters.

**Objectives:**

Using a rodent model of stress-induced hypophagia, we investigated if stress exposure augments food-related serotonin turnover and imbalances in measures of brain serotonin and dopamine activity in manners consistent with anxiousness toward food and restricted eating.

**Methods:**

Adult male F344 rats were conditioned to associate an audio cue with daily food over 2 weeks, after which half of the rats were exposed to a single episode of tail shocks (stress) or left undisturbed (nonstressed). All rats were killed 48 h later, during a control period, the food-associated cue, or a period of food access. Serotonin, dopamine, and norepinephrine, as well as metabolite concentrations, were assessed across brain regions comprising reward, emotion, and feeding circuits relevant to AN in acutely stressed and nonstressed rats using HPLC. Statistical significance level was 5%.

**Results:**

Stress-induced rat hypophagia paralleled an augmented serotonin turnover in response to the food-associated cue in the hypothalamus and hippocampus, as well as food access in the hypothalamus and cortical areas (all *P* < 0.05). Stress exposure increased the ratio of serotonin to dopamine metabolites across several brain areas, but the magnitude of this imbalance was further augmented during the food-associated cue and food access in the brainstem, hippocampus, and cortical areas (all *P* < 0.05). Finally, stress lowered norepinephrine concentrations by 18% in the hypothalamus (*P* < 0.05).

**Conclusions:**

The observed stress-induced changes to monoamine profiles in rats could have key implications for physiological states that contribute to restricted eating and may hold relevance for the development of AN precipitated by adverse life experiences.

## Introduction

Despite that an estimated 1% of all women and 0.5% of all men in the United States will have anorexia nervosa (AN) during their lifetime, much remains to be understood about this deadly eating disorder (1, 2). Nearly two-thirds of individuals with AN are also diagnosed with at least one anxiety disorder and maintain an anxious phenotype well after the eating disorder has been treated (3). Exposure to adverse life events (i.e., psychological traumas) is a critical risk factor linking both the development of anxiety disorders and AN (3–6). However, the neurobiology behind how adverse life events precipitate AN and its relation with anxiety is not well understood.

Eating-provoked anxious mood states associated with AN are postulated to be due, in part, to food-related exaggerated serotonin (5HT) concentrations in the brain [as reviewed in (7, 8)]. In fact, augmented 5HT concentrations may contribute to imbalances of monoamine neurotransmitter activity across the brain. For instance, disturbances to the balance of 5HT and dopamine (DA) are hypothesized to contribute to several traits that predict the development of AN, including mood disturbances, dysphoria, impulsivity, and cognitive inflexibility [as reviewed in (7, 9, 10)]. However, relatively little is known about how food-related 5HT activity may become augmented in a manner consistent with restricted eating or how it contributes to imbalances between brain monoamines.

Abnormal food-related 5HT activity associated with AN may be in response to prior exposure to adverse life events. Rodent models suggest exposure to a single episode of uncontrollable tail shocks (acute stress) can elicit anxiety-like behavior, as well as restricted eating (i.e., hypophagia) and impaired weight gain (11–18). Acute stress resembles psychological stress, such that when rats are given the ability to terminate each of the tail shocks by turning a small wheel, they do not develop anxiety-like behaviors compared with their yoked counterparts [as reviewed in (17–19)]. A culmination of over 5 decades of research suggests that abnormal 5HT neuron activity in the brainstem raphe nuclei is responsible for the development of rat anxiety-like behavior following exposure to acute stress [as reviewed in (17)]. Indeed, acute stress increases the sensitivity of 5HT neurons to future stimulation, causing an augmented release of 5HT during exposure to future stressors that would normally produce milder 5HT responses (17, 19–26). This acute stress–potentiated 5HT activity in brain areas that comprise motivation and emotion circuits is both necessary and sufficient to provoke anxiety-like behavior in rats [as reviewed in (17, 19)]. Despite a wealth of data supporting the involvement of excessive 5HT activity in the generation of anxiety behavior, the neural mechanisms behind the hypophagia following acute stress remain unclear.

One possibility is that food-related 5HT activity also becomes exaggerated following exposure to stressors of sufficient intensity to sensitize the activity of 5HT neurons. During feeding or exposure to food-associated cues, 5HT concentrations can become naturally stimulated in brain regions innervated by the raphe nuclei, including the hypothalamus, hippocampus, amygdala, and prefrontal cortex (26–32). This food-related 5HT activity may become augmented following exposure to acute stress. Acute stress–sensitized 5HT activity in reward and limbic structures can elicit anxiety-like behavior [as reviewed in (17, 19)]. Therefore, possible stress-induced, abnormal elevations in 5HT activity in these circuits related to food may provoke anxiety that contributes to food avoidance. However, the influence of acute stress on food-related 5HT activity in the rat brain and its possible contribution to monoamine imbalances across the brain remains unknown. Identifying the consequences of acute stress exposure on food-related 5HT activity in the rat brain could provide insights into the neurobiology of AN precipitated by adverse life experiences.

The purpose of this study was 2-fold: first, to investigate the persistence of rat hypophagia following exposure to acute tail shock stress, as existing research beyond a few days is limited (11, 12, 15), and, second, to investigate the influence of acute stress exposure on measures of 5HT turnover, as well as the balance of 5HT and DA activity across the brain during the presentation of an anticipatory food cue and a period of food access. To that end, the concentrations of 5HT, DA, and norepinephrine (NE), along with the DA metabolites 3,4-dihydrophenylacetic acid (DOPAC) and homovanillic acid (HVA), and the 5HT metabolite 5-hydroxindoleacetic acid (5HIAA) were measured in brain areas containing the prefrontal cortex, hypothalamus, striatum, hippocampus, hindbrain brainstem, and remaining caudal cortical region. These brain regions comprise limbic, reward, and feeding pathways within which abnormal monoamine activity may contribute to the development of anorexia-like behavior. The results of this study not only provide a comprehensive assessment of markers for monoaminergic activity across the rat brain associated with acute stress–induced hypophagia but may also have implications for the development of AN precipitated by adverse life experiences.

## Methods

### Animals

Adult male Fischer 344 rats (220–250 g upon arrival) were subjects, chosen per previous work documenting the hypophagia and sensitized 5HT neuron responses to the same stressor employed herein (11, 12, 20, 33). The first cohort of rats (*n* = 12) characterized long-term changes of weight, food consumption, and water ingestion following acute stress exposure. The second cohort of rats (*n* = 54) was used to analyze brain monoamine-related neurochemicals related to stress and food exposure (detailed below). Upon arrival, rats were housed 2–3 per cage, and no experimental manipulations took place during the first week. The base of their tails was marked with a colored permanent marker to distinguish rats in each cage. Food (ENVIGO, Tekland 2014, g/kg diet: crude protein, 143; fat, 40; carbohydrate, 480; crude fiber, 41; neutral detergent fiber, 180; ash, 47; AIN-93 mineral and vitamin mix) and water were provided ad libitum, except for experiment 2 (see below). Sample sizes were determined by power analyses completed on our previous work (20, 34–36). An unanticipated loss of 1 rat occurred in the second cohort and was not included in analysis. Rooms were controlled for temperature (21 ± 1°C) and photoperiod (12:12 light/dark) for the entire study. All procedures were approved by the Iowa State University Institutional Animal Care and Use Committee and adhered to NIH guidelines. Special care was taken to minimize animal discomfort during all procedures.

### Experiment 1: Persistence of acute stress–induced hypophagia

The purpose of this experiment was to examine the persistence of rat hypophagia following stress. On day 1, rat weight, food consumption, and water ingestion were recorded. The next day, rats were exposed to acute stress (stress, *n* = 6) or left undisturbed in home cages (no stress, *n* = 6) (see below for acute stress paradigm). Treatments were assigned randomly at cage level (i.e., each pair-housed rat experienced the same stress treatment). Rat body weight, food consumption, and water ingestion were recorded every third day for 21 d. The total weight of the food pellets and water in bottles from each cage was recorded and subtracted from the prior day's weights to obtain estimates of food consumption and water ingestion. These values were divided by the number of rats in each cage to estimate the average intake of food and water per animal.

#### Acute stress paradigm

Group-housed rats were assigned to receive uncontrollable tail shocks (stress) or no stress. The tail shock paradigm followed our previous publications (20, 34). Approximately 2–5 h into the light cycle, rats that received tail shocks were restrained in flat-bottom Plexiglas tubes with the tail protruding from the back where electrodes were placed to deliver 100, 5-s tail shocks on a variable 1-min intershock interval. Rats received 1.0-mA tail shocks for 50 min. Shock intensity was then increased to 1.5 mA for the remainder of the session. Rats that did not receive stress remained undisturbed in home cages in a different room during the tail shocks.

### Experiment 2: Influence of acute stress on food-related monoamine responses

This experiment tested the hypothesis that acute stress exposure (described above), which sensitizes the activity of brain 5HT neurons [as reviewed in (17–19)], augments brain 5HT turnover in association with food. All treatments detailed below were assigned randomly at the cage level (i.e., rats randomly assigned to each cage received the same treatment). Access to food pellets was restricted to only the dark photoperiod (i.e., when rats are awake and active) during the course of the entire experiment. At the onset of the dark photoperiod (i.e., lights-off), white noise (70 dB) was presented over a speaker continuously for 15 min immediately prior to the presentation of food pellets to the cage. The white noise served as an auditory cue to condition rats to the anticipation of food. Free access to food pellets continued for ∼12 h until the onset of the light photoperiod, after which all food pellets were removed from cages. After 14–18 d of cued food access, rats were exposed to a single episode of acute stress (stress, *n* = 26) or left undisturbed in their home cages (no stress, *n* = 27). Rats were exposed to stress in separate cohorts over 5 d (i.e., days 14–18), so that posteuthanasia tissue collection could occur rapidly and at the same times across groups detailed below. All rats were killed 48 h after acute stress, as it falls within the period of sensitized 5HT activity observed in rat brain reward and emotion structures [as reviewed in (17–19)].

Rats were killed in 3 groups. The first group, Food Cue (*n* = 9 no stress, *n* = 9 stress), was sampled after 12–15 min of the white noise cue but before food access. The Food Cue group was sampled to measure monoaminergic responses to food-related cues. The second group, Food Access (*n* = 9 no stress, *n* = 8 stress), was sampled after 30–35 min of food access (i.e., 45–50 min following onset of the food-associated cue). The Food Access group served to measure monoaminergic responses during feeding, corresponding with the period suggesting 5HT concentrations in the brain become altered by eating (27–29, 31). The third group, Inactive Control (*n* = 9 no stress, *n* = 9 stress), was sampled 7 h into the light cycle, a period of relative inactivity. Despite the possible influence of diurnal rhythms on neurochemical activity resulting from being killed hours before the other groups, this group served as a necessary control for baseline monoamine activity in the absence of feeding or the food-associated cue. One cage of nonstressed and stressed rats from each group was sampled each day.

#### High-performance liquid chromatography and analysis

One nonstressed rat and one stressed rat were killed simultaneously by rapid decapitation without sedation or anesthetic, and brains were quickly extracted. Brain regions containing the prefrontal cortex, remaining caudal cortical area, hypothalamus, cerebellum, striatum, hippocampus, and hindbrain brainstem areas were rapidly microdissected on a glass plate placed over ice [for detailed methods, see our previous publications (35, 36)]. Microdissected brain areas were placed in preweighed cryovials containing 0.2 M perchloric acid, flash-frozen with liquid nitrogen, weighed again to obtain sample weights, and then stored at –80°C in an ultra-low freezer until ultra-HPLC (UHPLC) processing. Detailed UHPLC methodology for assessing monoamine-related neurochemicals can be found in our previous publications [see (35, 36)].

Neurochemical measurements obtained from UHPLC were corrected for by gram weight of frozen tissue for each rat (i.e., μg of neurochemical/gram of tissue). The ratio of metabolite to respective neurotransmitter concentration [i.e., 5HIAA/5HT and (HVA + DOPAC)/DA] was calculated as a measure of neurotransmitter turnover in each sample condition [see (35–41)]. Moreover, imbalances between 5HT and DA activity in the brain may be of significance to AN (see Introduction). Therefore, the ratio of 5HT metabolites to DA metabolites (i.e., 5HIAA/HVA + DOPAC) was considered a marker for assessing the balance between 5HT and DA activity in each brain region, as when neurotransmitter activity is high, more neurotransmitter is released and consequently metabolized, thereby resulting in greater metabolite concentrations (41).

For the hypothalamus, prefrontal cortex, and cerebellum, DOPAC neurochemical concentrations fell below the lowest values of our UHPLC standards. For these brain regions, DA turnover was instead calculated as HVA/DA and balances of 5HT/DA metabolites as 5HIAA/HVA.

### Statistical analysis

The statistical unit for food consumption and water ingestion was cage. The statistical unit for body weight was rat. For the first experiment, group differences in rodent weight, food consumption, and water ingestion were assessed using a repeated-measures ANOVA. For the second experiment, weight change was calculated and compared between nonstressed and stressed rats by a *t*-test. Moreover, average daily food consumption for the 48 h prior to stress exposure and 24 h after stress exposure was measured at approximately the same time of day and compared by 2-factor ANOVAs. Markers of neurotransmitter turnover, the ratio of 5HT to DA metabolites, and individual neurochemical concentrations in each brain area were analyzed by 2-factor ANOVA, with stress and sample period (i.e., Inactive Control, Food Cue, food access) as factors. Statistically significant main effects of sample period, both sample period and stress, or interaction between these factors were followed by Tukey's Honestly Significant Difference post hoc analysis. Percentage differences between groups for only statistically significant post hoc tests were reported. Neurochemical concentration data points greater than 2 SD from the mean were excluded from statistical analysis. Statistical significance level was set to 0.05.

## Results

### Experiment 1: Persistence of acute stress–induced hypophagia

**FIGURE 1:**
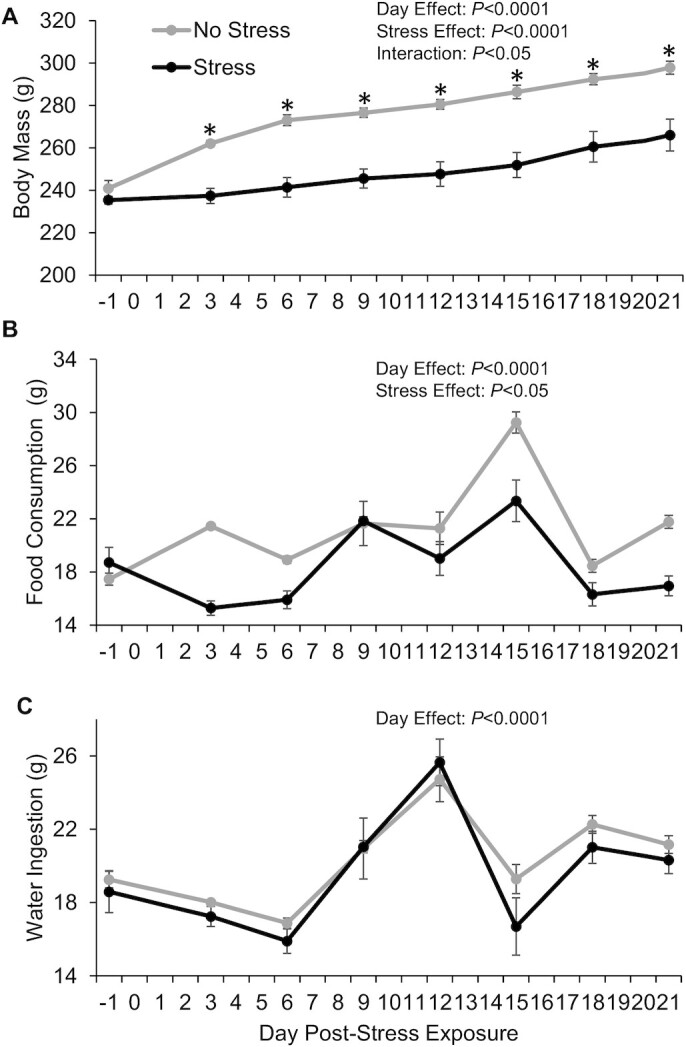
Hypophagia and reduced weight gain observed across 21 d in stressed compared with nonstressed rats for experiment 1. (A) Average body weight, (B) average daily food consumed, and (C) average daily water ingested (± SEM) for stressed and nonstressed rats before (i.e., day –1) to after (i.e., days 3–21) acute tail shock stress recorded on every third day. Note that acute stress exposure occurred on day 0. Data are means ± SEMs; *n* = 6 rats per group for body weight, *n* = 3 cages per group for food and water intake. *Different from control at each day (*P* < 0.05).

The data indicate that rats exposed to stress weighed ∼25–35 g less at each time point compared with their nonstressed counterparts over a 21-d period, despite starting at similar weights prior to stress (*P* = 0.025, interaction) (**Figure 1**A). The average amount of food consumed in stressed rats was lower than that of nonstressed rats on average by ∼4 g each day (*P =* 0.0091, main effect), but the total food intake varied by day (*P* < 0.0001, main effect) (Figure 1B). Finally, water ingestion over the same period varied across days (*P* < 0.0001, main effect) but was unaffected by stress exposure (Figure 1C). Together, these data suggest that stress exposure reduced food intake and body weight but not water ingestion for at least 21 d.

### Experiment 2: Influence of acute stress on food-related monoamine responses

#### Stress-induced changes in body weight and food consumption

**FIGURE 2:**
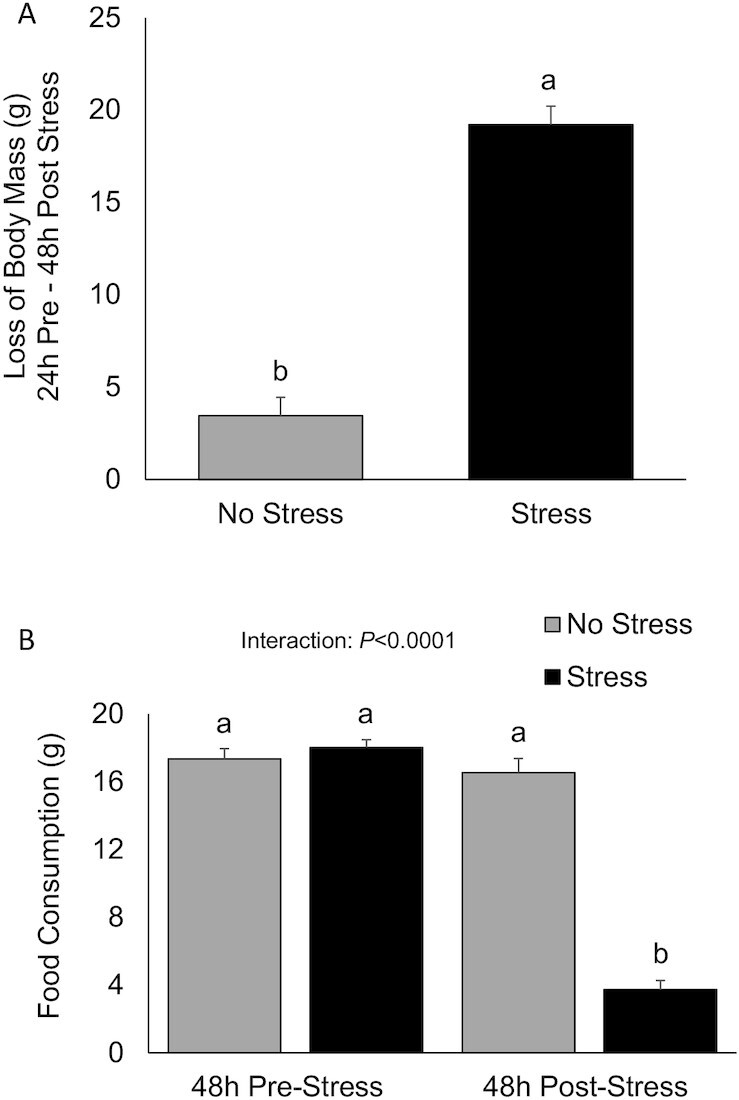
Hypophagia and reduced weight gain observed in stressed compared with nonstressed rats for experiment 2. (A) Loss of rat body weight measured 48 h after the day of acute tail shocks compared by *t*-test. (B) Change in daily weight of food consumed by rats measured during the 48 h before and 24 h after acute tail shock stress. Data are means ± SEMs; *n* = 26–27 rats per group for body weight, *n* = 13 cages per group for food intake. Note that rats were sampled for brain monoamine analyses 48 h after stress exposure. Groups that do not share a common letter differ significantly (*P* < 0.05).

Rats exposed to acute stress lost ∼15 g more body weight than nonstressed rats (*P* < 0.0001, *t*-test) (**Figure 2**A). Moreover, the amount of food consumed was ∼12–14 g lower in rats following stress exposure than prestress exposure, as well as compared with nonstressed rats before and after stress exposure (*P* < 0.0001, interaction) (Figure 2B).

#### Serotonin and dopamine turnover

**FIGURE 3:**
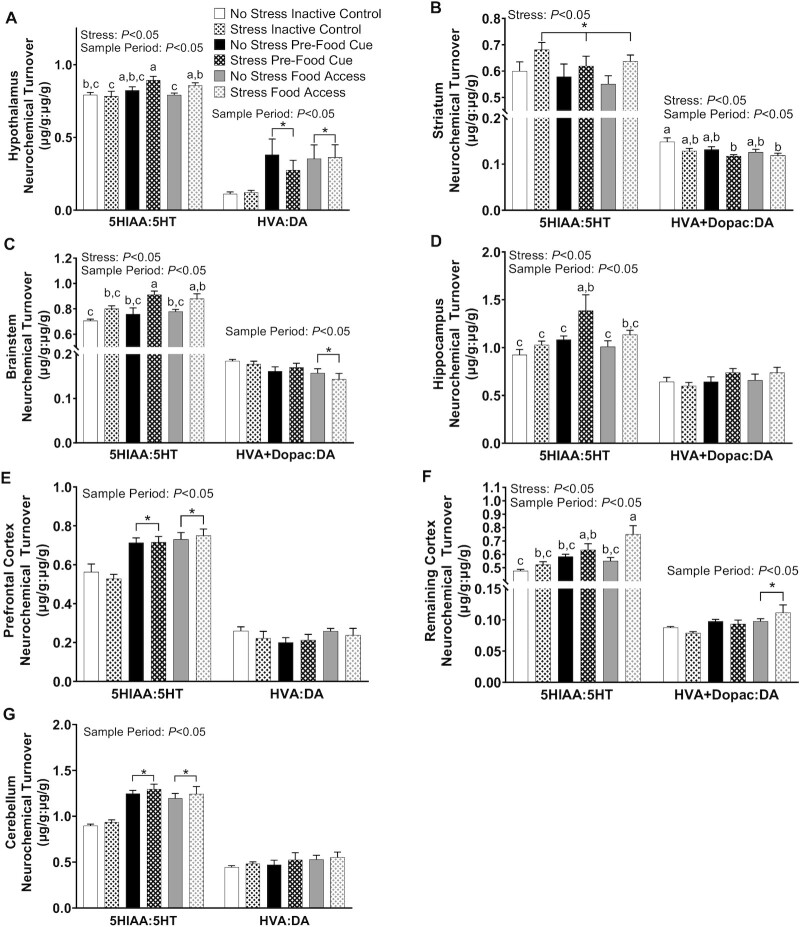
The influence of acute stress on measures of brain serotonin and dopamine turnover for rats sampled during the inactive period, a prefood cue, or period of food access for experiment 2. The ratio to 5HT and DA metabolite to neurotransmitter for nonstressed and stressed rats sampled during the inactive period (i.e., Inactive Control), the presentation of a food-associated cue (i.e., Food Cue), and after a period of food access (i.e., Food Access) in microdissected brain areas containing the (A) hypothalamus, (B) striatum, (C) brainstem, (D) hippocampus, (E) prefrontal cortex, and (F) cerebellum. Data are means ± SEMs; *n* = 8–9 rats per group. Groups that do not share a common letter differ significantly (*P* < 0.05). *Different from control (*P* < 0.05). DA, dopamine; DOPAC, 3,4-dihydrophenylacetic acid; HVA, homovanillic acid; 5HIAA, 5-hydroxindoleacetic acid; 5HT, serotonin.

*Hypothalamus*. Both sample period (*P* = 0.014, main effect) and stress exposure (*P* = 0.032, main effect) influenced 5HIAA/5HT in the hypothalamic areas, whereby 5HT turnover makers during the prefood cue and food access were mildly sensitized in stressed rats (**Figure 3**A). Indeed, rats exposed to stress and sampled during the prefood cue had greater 5HIAA/5HT by 12% and 15%, respectively, compared with nonstressed and stressed rats sampled during the inactive period, as well as 12% compared with nonstressed rats sampled during food access. Rats exposed to stress and sampled during food access had a greater 5HIAA/5HT by 10% compared with their nonstressed counterparts and by 12% compared with stressed rats sampled during the inactive period.

For HVA/DA, a significant main effect of sample period was observed (*P* = 0.0032, main effect), whereby rats sampled during the prefood cue and food access had a higher ratio than rats sampled during the inactive period by 95% and 101%, respectively (Figure 3A). However, exposure to stress did not influence DA turnover markers.

*Striatum*. Stress exposure, but not sample period, increased 5HIAA/5HT in the striatum by 11% (*P* = 0.018, main effect) (Figure 3B).

Markers of DA turnover in the striatum were lower in stressed rats (*P* = 0.0075, main effect), whereas the inactive period had higher concentrations than the other 2 sampling periods (*P* = 0.017, main effect) (Figure 3B). Indeed, nonstressed rats during the inactive period maintained 23% and 22% greater HVA + DOPAC/DA than stressed rats during prefood cues and food access, respectively.

*Brainstem*. 5HT turnover markers in the brainstem were elevated by stress exposure (*P* < 0.0001, main effect), whereas the inactive period had lower concentrations than the other 2 sampling periods (*P* = 0.0011, main effect) (Figure 3C). Post hoc analyses revealed stressed rats sampled during the prefood cue had greater 5HIAA:5HT than nonstressed rats sampled during the inactive period by 25%, prefood cue by 18%, and food access by 16%. Stressed rats sampled during food access had more 5HIAA/5HT than nonstressed rats sampled during the inactive period by 22%.

Finally, sample period, but not stress, influenced HVA + DOPAC/DA in the brainstem (*P* = 0.0057, main effect) (Figure 3C). Rats sampled during food access had an 18% lower HVA + DOPAC/DA than rats sampled during the inactive period.

*Hippocampus*. Sample period (*P* = 0.0084, main effect) and stress exposure (*P* < 0.010, main effect) influenced 5HIAA/5HT in the hippocampus, whereby stressed rats yielded the highest 5HT turnover during the prefood cue compared with all other groups (Figure 3D). Indeed, stressed rats sampled during the prefood cue presentation had greater 5HIAA/5HT by 26% compared with their nonstressed counterparts, by 40% and 30%, respectively, compared with nonstressed and stressed rats sampled during the inactive period, as well as by 31% compared with nonstressed rats sampled during food access.

Finally, hippocampal HVA + DOPAC/DA did not differ across sample period or as a result of stress exposure.

*Prefrontal cortex*. Sample period, but not stress, influenced prefrontal cortex 5HIAA/5HT (*P* < 0.0001, main effect) (Figure 3E). 5HIAA/5HT was lower during the inactive period than during both the prefood cue by 27% and food access by 30%. Neither sample period or stress influenced HVA/DA in the prefrontal cortex (Figure 3E).

*Remaining cortex*. Sample period (*P* = 0.0002, main effect) and stress exposure (*P* = 0.0011, main effect) influenced 5HIAA/5HT in the remaining cortex (Figure 3F), whereby 5HT turnover markers were elevated in stressed rats, and the inactive period saw lower concentrations compared with the other sampling periods (see Figure 3F). Stressed rats sampled during the prefood cue had a 28% greater 5HIAA/5HT than nonstressed rats sampled during the inactive period. In addition, stressed rats sampled during food access had a greater 5HIAA/5HT by 31% compared with their nonstressed counterparts, by 45% and 35%, respectively, compared with nonstressed and stressed rats in the inactive group, as well as by 25% compared with nonstressed rats sampled during the prefood cue.

Finally, HVA + DOPAC/DA was influenced by sample period, but not stress (*P* = 0.0023, main effect), in the remaining cortical areas (Figure 3F). Indeed, 23% lower HVA + DOPAC/DA was detected during the inactive period compared with during food access.

*Cerebellum*. Cerebellar 5HIAA/5HT was influenced by sample period, but not stress (*P* < 0.0001, main effect) (Figure 3G). Indeed, lower 5HT turnover concentrations were detected during the inactive period by 32% compared with prefood cue presentation and by 28% during food access. However, neither sample period nor stress exposure influenced cerebellar HVA + DOPAC/DA concentrations (Figure 3G).

#### Balance of 5HT and DA metabolites

**FIGURE 4:**
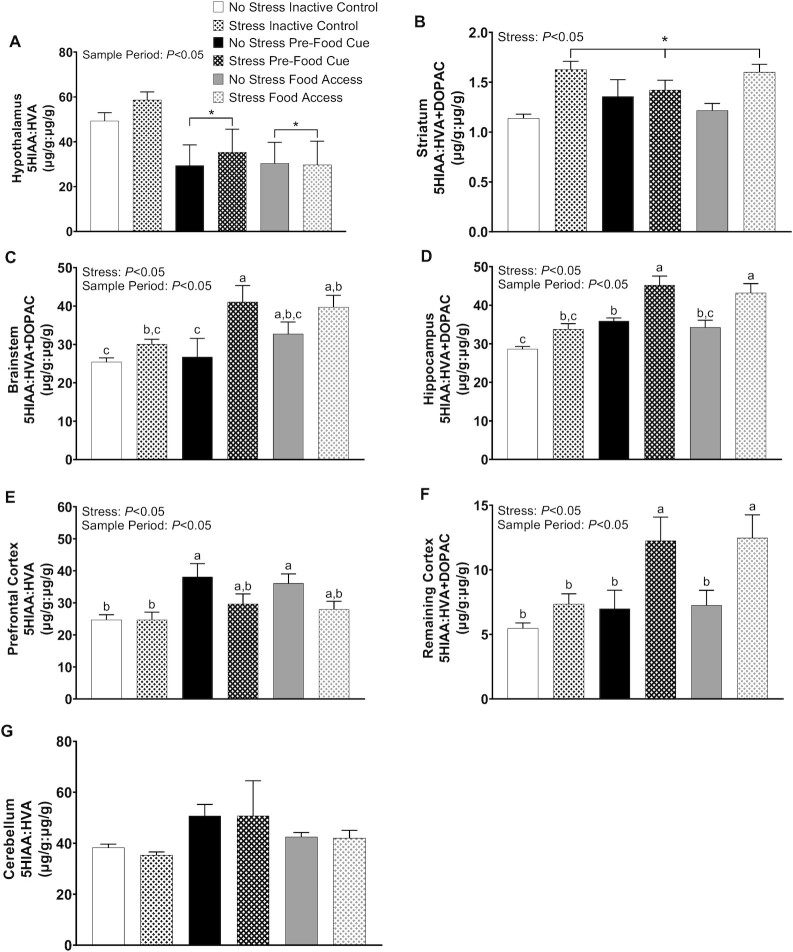
The influence of acute stress on the balance of 5HT and DA metabolites for rats sampled during the inactive period, a prefood cue, or period of food access for experiment 2. The ratio to 5HT metabolite to DA metabolite for nonstressed and stressed rats sampled during the inactive period (i.e., Inactive Control), the presentation of a food-associated cue (i.e., Food Cue), and after a period of food access (i.e., Food Access) in microdissected brain areas containing the (A) hypothalamus, (B) striatum, (C) brainstem, (D) hippocampus, (E) prefrontal cortex, and (F) cerebellum. Data are means ± SEMs; *n* = 8–9 rats per group. Groups that do not share a common letter differ significantly (*P* < 0.05). *Different from control (*P* < 0.05). DA, dopamine; DOPAC, 3,4-dihydrophenylacetic acid; HVA, homovanillic acid; 5HIAA, 5-hydroxindoleacetic acid; 5HT, serotonin.

*Hypothalamic area*. Sample period, but not stress, influenced 5HIAA/HVA (*P* = 0.0090, main effect) in the hypothalamic areas (**Figure 4**A). 5HIAA/HVA was higher during the inactive period than during the prefood cue by 50% and food access by 57%.

*Striatum. S*tress exposure increased striatal 5HIAA/HVA + DOPAC by 22% (*P* = 0.0003, main effect), but sample period was unaffected (Figure 4B).

*Brainstem*. Sample period (*P* = 0.035, main effect) and stress exposure (*P* = 0.0022, main effect) influenced 5HIAA/HVA + DOPAC in brainstem regions, whereby stress exposure elevated the 5HT metabolite compared with DA metabolites to the greater degree during the prefood cue and food access (Figure 4C). Indeed, rats exposed to stress and sampled during the prefood cue had a higher 5HIAA/HVA + DOPAC by 42% compared with their nonstressed counterparts, as well as by 47% and 34%, respectively, compared with nonstressed and stressed rats in the inactive condition. Moreover, rats exposed to stress and sampled during food access had a higher 5HIAA/HVA + DOPAC than nonstressed rats sampled in the inactive condition by 44% and during prefood cue by 38%.

*Hippocampus*. Stress exposure increased concentrations of hippocampal 5HT metabolite compared with DA metabolites across all sample periods (*P* < 0.0001, main effect), but the magnitude of the stress effect was marginally greater during prefood cue and food access periods (*P* < 0.0001, main effect) (Figure 4D). Post hoc analysis revealed that nonstressed rats sampled during the prefood cue had 23% higher 5HIAA/HVA + DOPAC than their counterparts sampled during the inactive period. Moreover, stressed rats sampled during the prefood cue had more 5HIAA/HVA + DOPAC than their nonstressed counterparts by 23% and nonstressed rats with food access by 28%, as well as nonstressed and stressed rats sampled during the inactive period by 45% and 29%, respectively. Finally, stressed rats sampled during food access had more 5HIAA/HVA + DOPAC than nonstressed rats sampled during the same period by 23% and during the prefood cue by 18%, as well as nonstressed and stressed rats sampled during the inactive period by 40% and 25%, respectively.

*Prefrontal cortex*. Sample period (*P =* 0.0059, main effect) and stress exposure (*P* = 0.024, main effect) influenced the ratio of 5HIAA/HVA in the prefrontal cortical area, whereby the increased concentration of 5HT to DA metabolites observed in the prefrontal cortex during prefood cue and food access was prevented by stress exposure (Figure 4E). Indeed, nonstressed rats sampled during the prefood cue had 42% greater 5HIAA/HVA compared with both nonstressed and stressed rats during the inactive period. Moreover, nonstressed rats sampled during food access had 37% more 5HIAA/HVA compared with rats that were both nonstressed and stressed sampled during the inactive period.

*Remaining cortex*. Stress exposure increased the concentration of 5HT/DA metabolites in the remaining cortex (*P* = 0.0004, main effect), but the magnitude of this effect was greater in rats sampled during the prefood cue and food access periods than inactive controls (*P =* 0.018, main effect) (Figure 4F). Indeed, stressed rats sampled during the prefood cue had greater 5HIAA/HVA + DOPAC than nonstressed rats sampled during the prefood cue by 55% and food access by 51%, as well as nonstressed and stressed rats sampled during the inactive periods by 76% and 50%, respectively. Similarly, stressed rats sampled during food access had greater 5HIAA/HVA + DOPAC than nonstressed rats sampled during the prefood cue by 56% and food access by 53%, as well as nonstressed and stressed rats sampled during the inactive periods by 77% and 52%, respectively.

*Cerebellum*. Neither stress nor sample period influenced 5HIAA/HVA in the cerebellum (Figure 4G).

Group comparisons for the individual neurochemicals used to calculate neurotransmitter turnover and the balance of 5HT and DA metabolites, as well as NE concentrations, are in the **Supplemental Figures 1–3**.

## Discussion

Exposure to adverse life events is a risk factor for the development of AN (3–6). Rodent models that display abnormal eating behaviors following stress exposure could provide insights into the neurophysiological conditions that mediate AN. This study yielded several findings that may have relevance for stress-induced AN. First, exposure to a single episode of acute stress resulted in long-lasting deficits of both food consumption and weight gain, persisting the duration of the 21-d study. Second, stress exposure augmented measures of 5HT turnover across several brain areas comprising motivation, emotion, and feeding circuits. The results also support an acute stress–induced sensitization of food-related 5HT turnover in the hypothalamus, hippocampus, and the remaining caudal cortical areas in manners consistent with greater food avoidance or restricted eating. Third, an analysis of 5HT/DA metabolites provides evidence that 5HT activity may be elevated compared with DA following acute stress in multiple brain areas, including the striatum, brainstem, hippocampus, and remaining cortical structures. These findings are generally consistent with hypotheses suggesting that competition between the opposing activities of 5HT and DA in the brain may contribute to traits associated with risk of developing anorexic behaviors. Together, these data may have importance for understanding the neurobiology of acute stress–induced eating disturbances, which could be of relevance for the development of AN precipitated by adverse life events.

An unanticipated finding of this study was the persistence of rat hypophagia and reduced body weight following exposure to acute stress, which lasted for the entire 21-d study. This was particularly surprising because rat feeding and weight gain deficits continued for much longer than the 72-h period after acute stress exposure, during which many of the anxiety- and depression-like behaviors dissipate [as reviewed in (17–19)]. These data suggest that the neural circuits involved in hypophagia may contain distinctions from those that contribute to the development of anxiety- and depression-like behavior following acute stress exposure, which have been comprehensively dissected [as reviewed in (17, 19)]. It is also worth noting that stressed rats did not display deficits in all consummatory behavior, as food consumption decreased, but water ingestion remained similar to unstressed rats. Therefore, perhaps the caloric value of the chow or the effects that bioactive dietary components have on the body contribute to its restriction in acutely stressed animals. The latter notion is consistent with the hypothesis that individuals with AN avoid food in an effort to attenuate the anxiety eliciting 5HT activity, by lowering 5HT synthesis through the restriction of food-derived tryptophan (i.e., “serotonin over activity hypothesis”) [as reviewed in (7, 42, 43)]. Indeed, evidence suggests tryptophan depletion can reduce anxious traits in anorexic individuals (43). In the current study, stress-induced exaggerated 5HT turnover was observed across several rat brain regions, which is an established cause of anxiety-like behaviors in rats [as reviewed in (17–19)].

Decades of research have implicated abnormal monoaminergic activity in the development of AN, as well as many other stress-related psychiatric conditions. The observations that 5HT activity is exaggerated in individuals at risk for AN have led to the development of several theories regarding this particular monoamine's involvement in the eating disorder (7, 43–45). Indeed, augmented 5HT activity in individuals with AN has been postulated to be a cause of anxious states promoting restricted eating, a neurochemical response contributing to food-specific dysphoria, and physiological states driving traits that underlie the development of AN [as reviewed in (7, 8, 45)]. Perhaps unifying these different hypotheses is the likelihood that features of AN may be mediated, in part, by distinct 5HT responses across brain regions following exposure to adverse life events. For instance, the current study found evidence for a poststress-induced augmentation of 5HT turnover in the striatum, brainstem, hippocampus, and caudal cortical areas. Such stress-augmented 5HT activity could contribute to a heightened state of anxiety (17, 19) and, perhaps, mediate reduced eating in an effort to lower 5HT synthesis (42).

Some evidence was also found supporting a stress-sensitized 5HT response specifically to food-associated cues and feeding in the hypothalamus, during food cue presentation in the hippocampus, and during feeding in the caudal cortical areas. Stress-sensitized 5HT activity in relation to food could contribute more precisely to dysphoric feelings toward food, as opposed to a heightened generally anxious state (7, 8, 44). For instance, exposure to food cues and satiety can naturally stimulate 5HT activity in the hypothalamus (28–31). Abnormally elevated food-specific 5HT activity in the hypothalamus might evoke a stronger stress response, as there is a positive relation between 5HT activity in this brain area and the release of stress hormones (46–48). Thus, an elevated stress response may become associated with food and consequently contribute to the development of food-related dysphoria. This hypothesis could be worth further investigation by future studies, as it is consistent with observations that activity in neural circuits involved in the association of euphoric feelings with pleasurable stimuli may instead become associated with dysphoria in individuals with AN (49). It also may be of interest that the hippocampus, a brain region widely implicated in associative learning [for review see (50, 51)], displayed augmented 5HT turnover during a food-associated cue, especially when considering that sufficiently elevated 5HT activity in the hippocampus is implicated in anxiety and avoidance behavior (52–55). However, the possible links between stress-exaggerated 5HT activity and food dysphoria certainly cannot be concluded from the current data. Future investigations employing pharmacological interventions targeting stress-elevated 5HT responses to food in the rat hypothalamus and hippocampus could provide a more definitive role for their possible involvement in food-specific dysphoria.

Augmented 5HT activity following acute stress exposure could drive imbalances between the activities of 5HT and DA in the brain, which have been hypothesized to contribute to traits that increase the risk of developing AN, including harm avoidance, cognitive inflexibility, food dysphoria, and so on [as reviewed in 7, 9,10]. In fact, 5HT is suggested to serve as a substrate of aversive signaling that opposes the DA-associated appetitive signaling in the brain (7, 9, 56, 57). Although the interactions of 5HT and DA systems across the brain are complex and influence many modulatory systems that in turn can affect behavior, some evidence suggests that heightened 5HT activity can dampen the release of DA in reward circuits (20, 56, 58–60). These findings are consistent with observations in the current study, whereby stress exposure potentiated measures of 5HT turnover in the striatum and brainstem and decreased measures of DA turnover in the striatum. Additional evidence for a stress-induced imbalance of 5HT and DA activity was observed across several brain areas, including the striatum, brainstem, hippocampus, and remaining caudal cortical areas. Interestingly, some of these stress-altered ratios were observed to a mildly greater degree during the food cue or feeding periods, compared with the inactive period, suggesting a diurnal or perhaps food-related interaction. The series of behavioral deficits that develop following exposure to the uncontrollable tail shock paradigm parallel some of the traits that are also associated with human risk for AN, such as anxiety, impaired appetitive behaviors, impulsivity, and cognitive inflexibility (21, 22). Therefore, this rodent model could serve as a useful tool to investigate the possible impact of pharmacologically restoring the poststress balance of 5HT and DA on hypophagia, as well as other traits underlying the risk of AN development.

In summary, these data provide evidence for a possible mechanism by which adverse life experiences alter monoaminergic responses to food in ways that may contribute to food dysphoria and restricted eating. Support for a stress-sensitized 5HT response to food was observed in the rat hypothalamus, hippocampus, and caudal cortical areas in association with restricted eating, which could be pertinent to the development of food-related dysphoria (7, 43, 45). Furthermore, stress-induced disturbances to the ratios of 5HT and DA metabolites in the striatum, brainstem, hippocampus, prefrontal cortex, and remaining cortical areas may represent imbalances between the activities of these two neurochemicals, which could have relevance for the neurobiological underpinnings of traits associated with AN risk [as reviewed in (7, 9, 10)]. The observations in the current study are consistent with several modern theories about monoaminergic involvement in AN [as reviewed in (7–9, 42, 56, 57)]. Thus, this rodent model could represent a useful preclinical screening tool of novel pharmacological approaches targeting monoaminergic systems in AN recovery. These topics warrant further investigation, as they could be pertinent to the etiology and treatment of AN.

## Supplementary Material

nxab298_Supplemental_File

## Notes

Supported by the Iowa State University Experiment Station.

Author disclosures: The authors report no conflicts of interest.

Supplemental Figures 1–3 are available from the “Supplementary data” link in the online posting of the article and from the same link in the online table of contents at https://academic.oup.com/jn/.

Abbreviations used: AN, anorexia nervosa; DA, dopamine; DOPAC, 3,4-dihydrophenylacetic acid; HVA, homovanillic acid; NE, norepinephrine; UHPLC, ultra-HPLC; 5HIAA, 5-hydroxindoleacetic acid; 5HT, serotonin.
