# Supplementary material for: Acute Stress Exposure Alters Food-Related Brain Monoaminergic Profiles in a Rat Model of Anorexia
Source: J Nutr. 2021 Sep 14;151(12):3617–27. doi: 10.1093/jn/nxab298 (PMC8643607; doi:10.1093/jn/nxab298)
Supplement: nxab298_Supplemental_File [file nxab298_supplemental_file.docx]

Acute stress exposure alters food-related brain monoaminergic profiles in a rat model of anorexia

Reed et al.

Online Supplementary Material


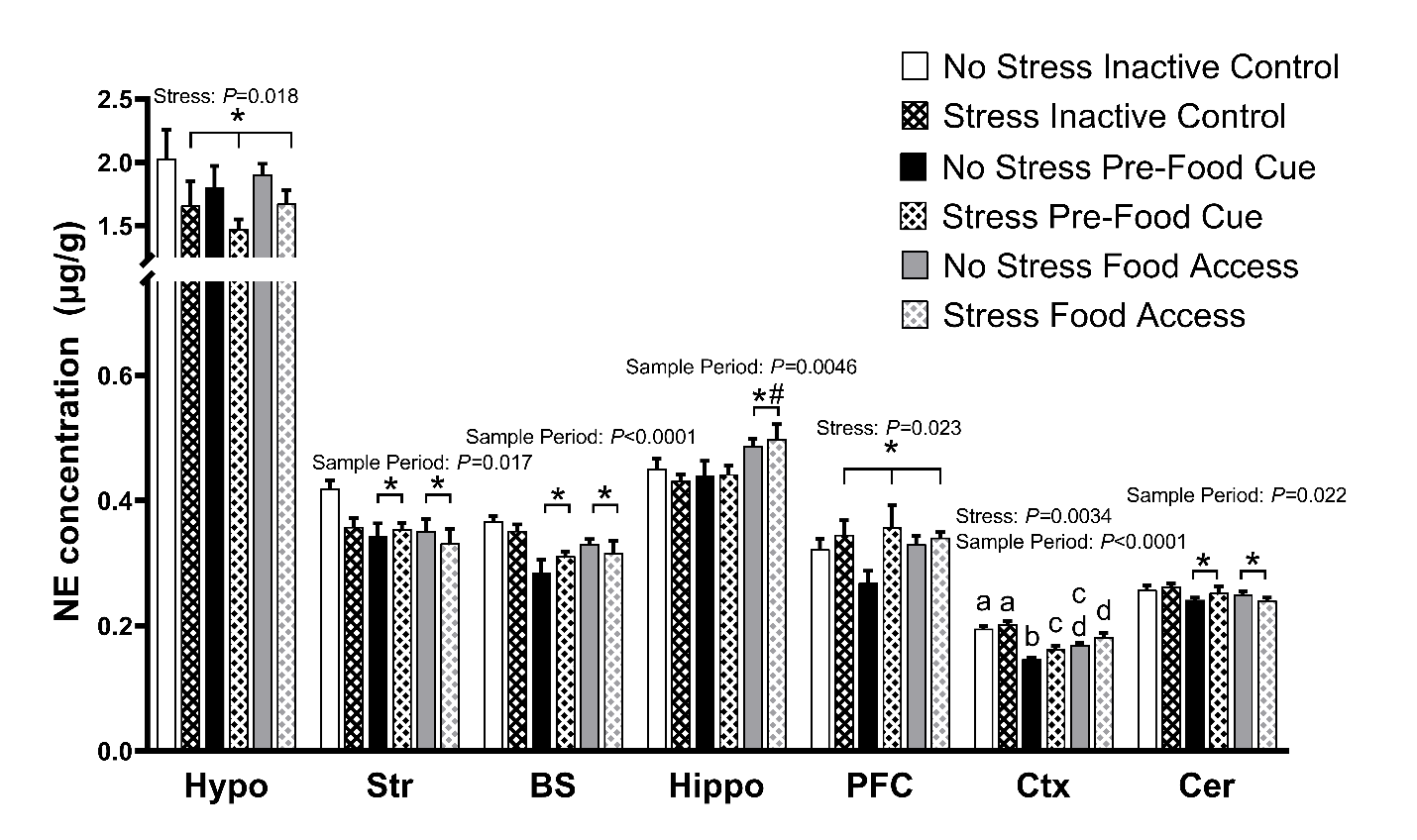


**Supplementary Figure 1**: **The influence of acute stress on norepinephrine concentrations across the brain of rats sampled during the inactive period, a pre-food cue, or period of food access for experiment 2.** NE concentrations of non-stressed and stressed rats sampled during the inactive period (i.e. Inactive Control), the presentation of a food-associated cue (i.e. Food Cue), and after a period of food access (i.e. Food Access) in microdissected brain areas containing the Hypothalamus (Hypo), Striatum (Str), Brainstem (BS), Hippocampus (Hippo), Pre-frontal Cortex (PFC), and Cerebellum (CER). Data are means (+/-SEM); *n* =8-9 rats per group. Groups that do not share a common letter differ significantly (*P* < 0.05). *Different from control (*P* < 0.05). #Different from Pre-Food cue (*P* < 0.05).


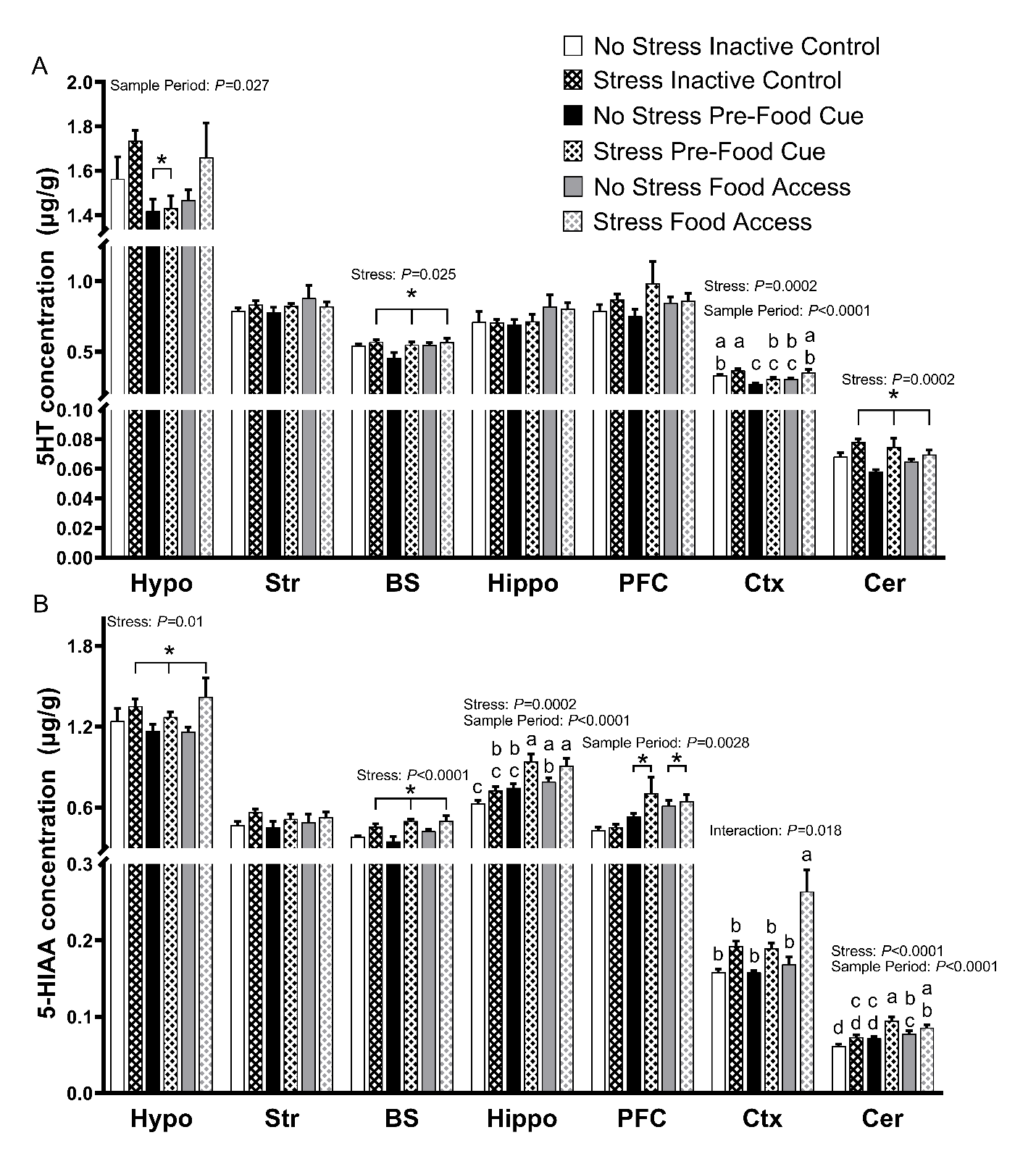


**Supplementary Figure 2**: **The influence of acute stress on serotonin-related neurochemical concentrations across the brain of rats sampled during the inactive period, a pre-food cue, or period of food access for experiment 2. (A)** 5HT concentrations and **(B)** 5HIAA concentrations of non-stressed and stressed rats sampled during the inactive period (i.e. Inactive Control), the presentation of a food-associated cue (i.e. Pre-Food), and after a period of food access (i.e. Post-Food) in microdissected brain areas containing the Hypothalamus (Hypo), Striatum (Str), Brainstem (BS), Hippocampus (Hippo), Pre-frontal Cortex (PFC), and Cerebellum (CER). Data are means (+/-SEM); *n* =8-9 rats per group. Groups compared by ANOVA denoting main effects of both stress and sample condition where letters present, brackets denoting main effects of either stress or sample condition when symbols present (*P* < 0.05). Groups that do not share a common letter differ significantly (*P* < 0.05). *Different from control (*P* < 0.05).


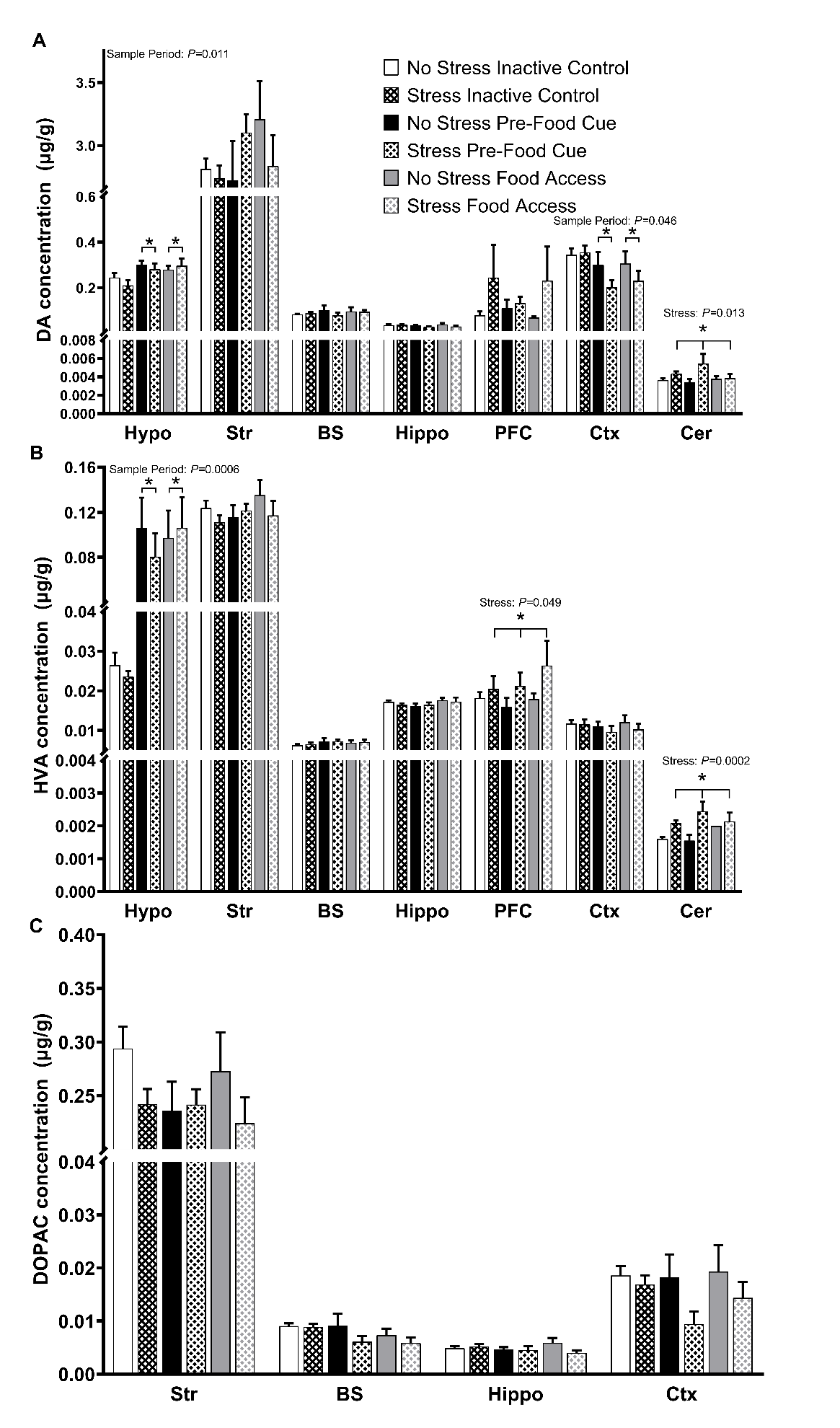


**Supplementary Figure 3**: **The influence of acute stress on dopamine-related neurochemical concentrations across the brain of rats sampled during the inactive period, a pre-food cue, or period of food access for experiment 2. (A)** DA concentrations, **(B)** HVA concentrations, and **(C)** DOPAC concentrations of non-stressed and stressed rats sampled during the inactive period (i.e. Inactive Control), the presentation of a food-associated cue (i.e. Pre-Food), and after a period of food access (i.e. Post-Food) in microdissected brain areas containing the Hypothalamus (Hypo), Striatum (Str), Brainstem (BS), Hippocampus (Hippo), Pre-frontal Cortex (PFC), and Cerebellum (CER). Data are means (+/-SEM); *n* =8-9 rats per group. Groups compared by ANOVA denoting main effects of both stress and sample condition where letters present, brackets denoting main effects of either stress or sample condition when symbols present (*P* < 0.05). *Different from control (*P* < 0.05).
